# Genome-Wide Identification and Expression Profiling of the *DOG1*-like Genes in Radish (*Raphanus sativus* L.)

**DOI:** 10.3390/ijms27156761

**Published:** 2026-07-28

**Authors:** Minyan Mai, Jinglei Wang, Zhijie Liu, Wuhong Wang, Haijiao Hu, Qingzhen Wei, Yaqin Yan, Chonglai Bao, Tianhua Hu

**Affiliations:** 1 Institute of Vegetable Crops, Zhejiang Academy of Agricultural Sciences, Hangzhou 310021, China; maiminyan2003@163.com (M.M.); angjinglei@zaas.ac.cn (J.W.); liuzhijie2025@126.com (Z.L.); wangwh@zaas.ac.cn (W.W.); huhj@zaas.ac.cn (H.H.); weiqz@zaas.ac.cn (Q.W.); yanyq@zaas.ac.cn (Y.Y.); 2 College of Horticulture, Zhejiang A&F University, Hangzhou 311300, China

**Keywords:** *DOG1*, radish, seed dormancy, gene family, expression profiling

## Abstract

Seed dormancy is a vital adaptive mechanism regulated by the DELAY OF GERMINATION (*DOG*) gene family. Here, we present the first systematic genome-wide identification and expression profiling of the *DOG* gene family (*RsDOGs*) in radish (*Raphanus sativus*). Comprehensive analysis of physicochemical properties, chromosomal distribution, and phylogeny revealed strong evolutionary conservation alongside functional divergence. Notably, several RsDOG proteins harbor natural fusions of the DOG1 domain with a bZIP domain, suggesting potential transcription factor activity and novel regulatory functions. Promoter analysis identified abundant ABA-responsive and abiotic stress-responsive cis-acting elements. Quantitative real-time PCR (qRT-PCR) profiling across contrasting cultivars—dormant variety Rs275 and non-dormant variety Rs100—at 4 h and 28 h post-imbibition revealed distinct differential expression patterns. In particular, TRs0x5c022182.1 exhibited high expression levels in dormant seeds, suggesting its potential involvement in seed dormancy regulation in radish. This study provides key insights into the structural evolution and expression dynamics of *RsDOG* genes, laying a solid foundation for molecular breeding aimed at optimizing seed germination traits.

## 1. Introduction

Seed dormancy is a vital adaptive mechanism that has evolved in plants over long evolutionary periods, enabling seeds to germinate under favorable environmental conditions and thereby enhancing plant survival [1]. Among the numerous genes involved in regulating seed dormancy and germination, the *DOG1* (Delay of Germination) gene family plays a central regulatory role [2]. Proteins encoded by these DOG1-like genes are primarily characterized by a highly conserved DOG1 domain, which serves as the defining structural feature uniting these members into a specific gene family. *DOG*1 was initially identified as a major quantitative trait locus (QTL) controlling seed dormancy in *Arabidopsis thaliana* [3]. Studies have demonstrated that *DOG1* functions in concert with the abscisic acid (ABA) signaling pathway to regulate seed dormancy [4] and acts as a negative regulator affecting gibberellin (GA) metabolism [2]. Furthermore, *DOG1* expression is temperature-sensitive and can prevent seed germination under unfavorable environmental conditions through temperature-sensing mechanisms [2]. With further investigation, accumulating evidence indicates that the *DOG1* family not only participates in dormancy regulation but also plays multiple roles in various plant growth, development, and stress tolerance processes, including the control of early flowering time and drought tolerance [3]. In the model species *Arabidopsis thaliana*, a total of four *DOG1* family members have been identified. The *DOG1*-like genes were retrieved and verified through sequence alignments and conserved domain analysis utilizing the TAIR and InterPro databases.

Radish (*Raphanus sativus*) is a globally cultivated and economically important Brassicaceae root vegetable crop [4]. Seed dormancy characteristics, germination efficiency, and tolerance to abiotic stresses are directly linked to radish cultivar improvement and agricultural production [4]. Although the *DOG1* gene family has been extensively identified and functionally characterized in various plant species, including *Arabidopsis*, rice, wheat [5], and moso bamboo [6], genome-wide identification and structural evolutionary analysis of the *DOG1* gene family in radish remain lacking.

To investigate the evolutionary mechanisms and potential functions of *DOG1* genes in radish, this study systematically identified members of the radish *DOG1* gene family (*RsDOG1s*) at the genome-wide level based on the radish whole-genome sequence [7]. In total, 25 DOG1 genes were identified in the radish genome. We comprehensively analyzed the physicochemical properties, chromosomal localization, and phylogenetic relationships of the *RsDOG1* gene family, with a focus on characterizing gene structure and conserved domain features. Notably, we identified a bZIP domain fusion phenomenon in radish DOG1 proteins, similar to that reported in moso bamboo [6]. Moreover, through promoter cis-acting element analysis, we further elucidated the potential regulatory networks through which *RsDOG1s* respond to phytohormones such as ABA and abiotic stresses. This study not only provides insights into the evolutionary patterns of the *DOG1* gene family in dicotyledonous plants but also establishes a theoretical foundation for future functional validation and molecular breeding of stress tolerance and dormancy-related genes in radish.

## 2. Results

### 2.1. Genome-Wide Identification of the Radish DOG1 Gene Family and Analysis of the Physicochemical Properties of Putative Proteins

A total of 25 radish *DOG1-like* genes encoding proteins containing complete conserved DOG1 domains were identified. Physicochemical property analysis (Table 1) revealed that the amino acid lengths of the 25 RsDOG1 proteins ranged from 237 aa (*TRs0x5c026231.1*) to 799 aa (*TRs0x6c030499.1*), with molecular weights between 27.20 kDa and 87.19 kDa. The theoretical isoelectric points (pI) exhibited a broad range from 5.07 (*TRs0x2c007663.1*) to 9.13 (*TRs0x7c034547.1*), encompassing both acidic and basic proteins, which may be associated with their functional differentiation in distinct intracellular microenvironments. Regarding the instability index, except for *TRs0x1c004725.1* (39.95), which was below 40 and thus classified as a stable protein, the remaining 24 RsDOG1 proteins had instability indices exceeding 40 (up to 69.8), indicating that the vast majority of RsDOG1 proteins are unstable in vitro. The aliphatic index of all the RsDOG1 proteins ranged from 73.71 to 94.31, while the grand average of hydropathicity (GRAVY) values was all negative (−0.187 to −0.717), demonstrating that all members of the radish DOG1 family are hydrophilic proteins.

### 2.2. Chromosomal Distribution Characteristics of Radish DOG1 Genes

**Figure 1:**
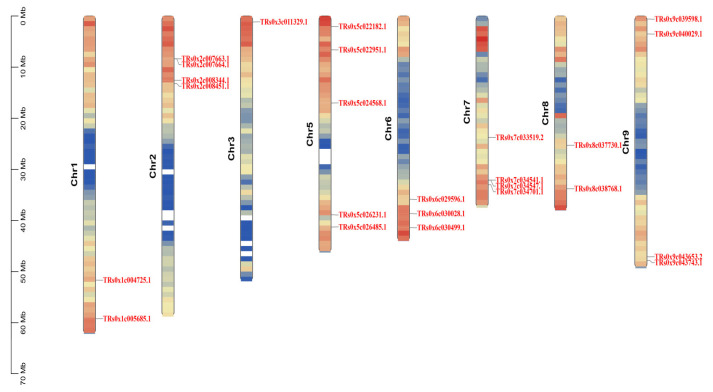
Chromosomal distribution of the radish *DOG1* gene family. Physical localization information of *RsDOG1* genes on the radish chromosomes (excluding Chr4) is indicated in red. Gene density is shown as a color gradient from blue (low density) to red (high density).

Based on the radish genome annotation information, we performed chromosomal localization of the 25 *RsDOG1* genes (Figure 1). The results showed that *RsDOG1* genes are unevenly distributed across eight chromosomes of radish, with no members mapped to Chr4. Specifically, Chr5 harbored the highest number of *RsDOG1* genes, with five members; Chr2, Chr7, and Chr9 each contained four genes; Chr6 carried three genes; Chr1 and Chr8 each had two genes; and Chr3 harbored the fewest, with only a single gene. This uneven distribution pattern has been similarly reported in the *DOG1* families of other species such as peanut [8]. Furthermore, on chromosomes Chr2 and Chr7, several *RsDOG1* genes were found to be highly clustered in physical proximity, suggesting that tandem duplication events may be a major driving force for gene expansion during the evolution of the radish *DOG1* gene family [9].

### 2.3. Phylogenetic Analysis of the Radish DOG1 Family with Arabidopsis

**Figure 2:**
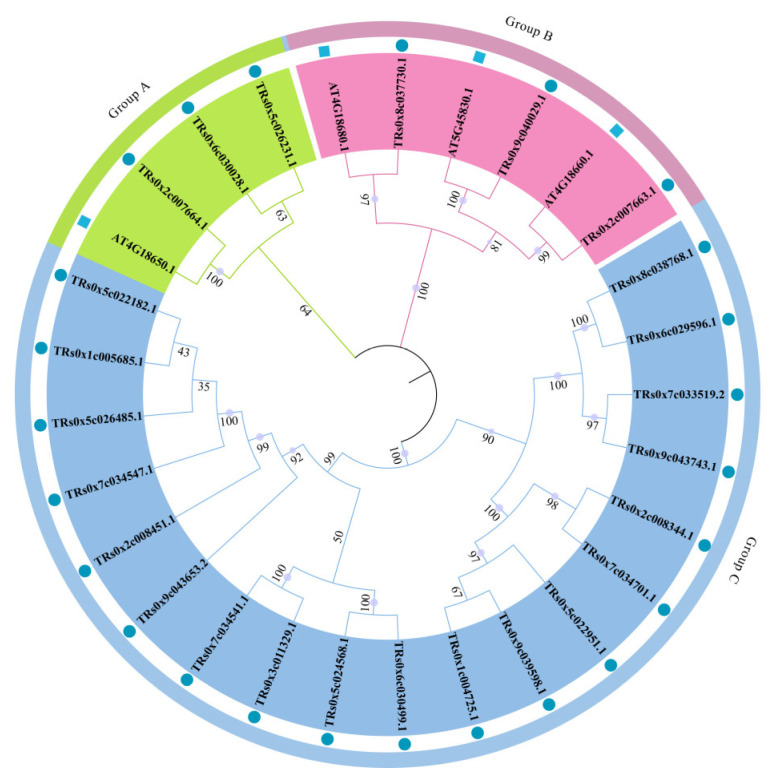
Unrooted phylogenetic tree of the *DOG1* family proteins from radish and Arabidopsis thaliana. The green, pink, and blue sectors represent the three major clades: Group A, Group B, and Group C, respectively. Dark blue circles indicate radish RsDOG1 proteins, and light blue squares represent Arabidopsis AtDOG1 proteins.

Figure 2 presents the phylogenetic analysis of the DOG1 family proteins from radish and *Arabidopsis thaliana*. Phylogenetic tree construction facilitates the elucidation of evolutionary relationships within the radish *DOG1* gene family and provides important clues for understanding their functional diversification. To explore these evolutionary relationships, an unrooted phylogenetic tree was constructed incorporating 25 radish RsDOG1 proteins and 4 Arabidopsis AtDOG1 proteins. On the tree, all the radish *RsDOG1* members are visually highlighted with dark blue circles, while Arabidopsis members are designated with light blue squares. Based on the topological structure, these proteins were clearly divided into three major clades: Group A, Group B, and Group C.

Specifically, Group A contained three radish proteins and one *Arabidopsis* protein (*AT4G18650.1*). Group B displayed an interleaved clustering pattern, comprising three radish proteins and three Arabidopsis members (*AT4G18680.1*, *AT5G45830.1*, and *AT4G18660.1*). In contrast, Group C consisted exclusively of the remaining 19 radish RsDOG1 proteins, forming a distinct cluster without any Arabidopsis orthologs. The close phylogenetic relationship and clustering patterns of the radish and Arabidopsis members within Groups A and B strongly confirm that radish shares a high degree of evolutionary affinity with Arabidopsis, both belonging to the dicotyledonous Brassicaceae family.

### 2.4. Gene Structure, Conserved Motif, and Domain Characteristics of Radish DOG1 Genes

**Figure 3:**
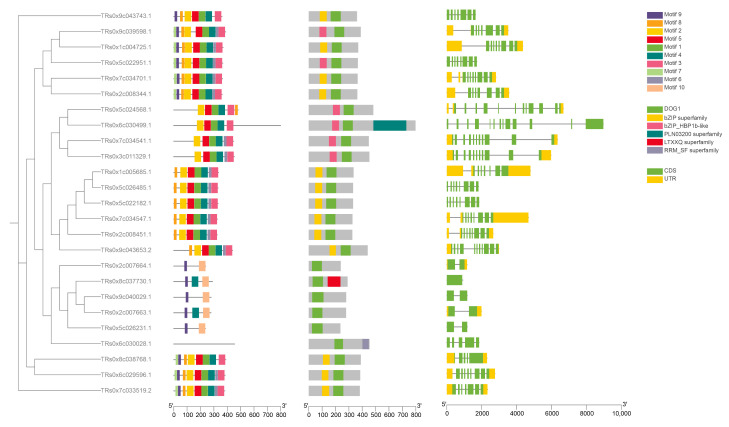
Integrated analysis of the phylogenetic tree, conserved motifs, conserved domains, and gene structures of the radish *DOG1* gene family. From left to right: phylogenetic tree, distribution of protein conserved domains, conserved motif composition, and exon–intron gene structure (CDS and UTR).

Through integrated analysis of the phylogeny, conserved motifs, conserved domains, and gene structures of *RsDOG1* genes (Figure 3), we found that the *RsDOG1* family exhibits a high degree of structural conservation accompanied by certain divergence. Regarding conserved domains, all 25 members contained the core *DOG1* domain. Notably, in addition to the DOG1 domain, several RsDOG1 proteins also harbored specialized domains belonging to the bZIP superfamily, bZIP_HBP1b-like, PLN03200 superfamily, LTXXQ superfamily, or RRM_SF superfamily. This finding is highly consistent with results from moso bamboo, where the fusion of DOG1 proteins with bZIP transcription factor domains was also confirmed [6], suggesting that these RsDOG1 proteins may possess the DNA-binding function of bZIP transcription factors and participate in more complex stress responses or hormone signal transduction pathways [10].

In terms of conserved motifs, a total of 10 motifs were predicted, with most members sharing Motif 1, Motif 2, Motif 4, and Motif 6. Members clustered within the same subclade of the phylogenetic tree tended to exhibit highly similar motif arrangement patterns [11]. Regarding exon–intron gene structure, *RsDOG1* genes displayed diversity: most genes exhibited relatively simple structures (containing 1–3 exons, with green boxes representing CDS and yellow boxes representing UTR), similar to the structure of *Arabidopsis AtDOG1*; whereas several individual genes (e.g., *TRs0x3c011329.1* and *TRs0x6c030499.1*) possessed longer sequences and more introns, suggesting that domain acquisition or sequence insertion may have occurred during evolution [12].

### 2.5. Characteristics of Cis-Acting Elements in RsDOG1 Promoters

**Figure 4:**
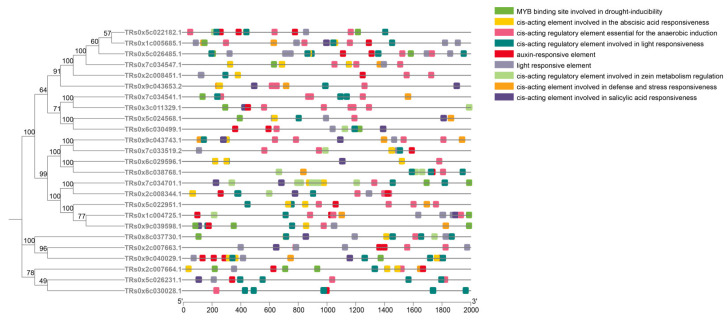
Distribution of cis-acting elements in the promoter regions (2000 bp upstream) of the radish *DOG1* gene family. Different colored rectangular blocks represent key regulatory elements responsive to various phytohormones and environmental stresses.

Cis-acting elements in promoter regions play a decisive role in the transcriptional regulation of genes (Figure 4). In terms of hormone responsiveness, the cis-acting element involved in abscisic acid responsiveness was the most widely distributed and most abundant element, present in the promoters of nearly all the *RsDOG1* genes. Additionally, cis-acting elements involved in salicylic acid responsiveness and auxin-responsive elements were also widely identified across multiple members. These findings indicate that *RsDOG1* genes are deeply involved in hormone-mediated signaling pathways, particularly ABA signaling, supporting the central role of *DOG1* in ABA-dependent seed dormancy regulation [13].

In terms of stress and environmental responsiveness, a large number of genes contained MYB binding sites involved in drought-inducibility, cis-acting regulatory elements essential for anaerobic induction, and cis-acting elements involved in defense and stress responsiveness. Furthermore, light-responsive elements, including both the cis-acting regulatory elements involved in light responsiveness and general light-responsive elements, were widely distributed across all family members [14]. The distribution characteristics of these cis-acting elements indicate that *RsDOG1* genes not only function in radish seed dormancy and germination but may also serve as environmental sensors, broadly participating in the adaptive responses of radish to abiotic stresses such as light, drought, and hypoxia [5].

### 2.6. Quantitative Real-Time Expression Analysis

**Figure 5:**
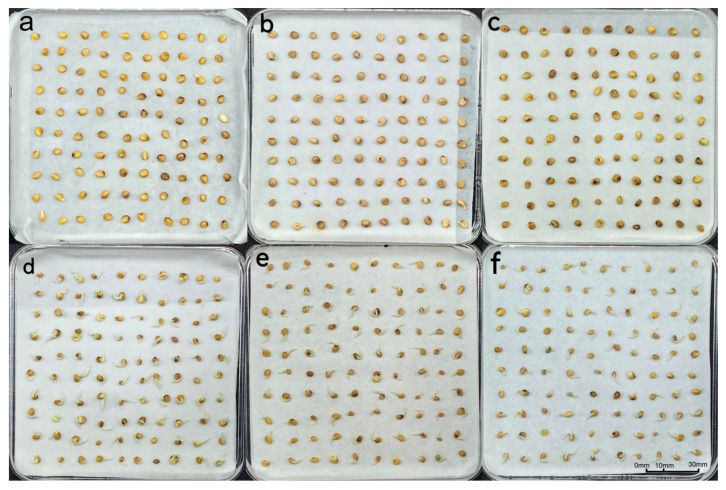
Phenotypic characteristics of dormant (Rs275) and non-dormant (Rs100) radish seeds after 24 h, with three replicates. Each square culture dish measures 130 mm × 130 mm, with individual seeds uniformly spaced at intervals of approximately 10 mm to serve as an internal scale reference. (**a**–**c**) Phenotypic records of the dormant variety Rs275 at 24 h after germination: (**a**) germination rate 11%, (**b**) germination rate 20%, (**c**) germination rate 12%. (**d**–**f**) Phenotypic records of the non-dormant variety Rs100 at 24 h after germination: (**d**) germination rate 98%, (**e**) germination rate 99%, (**f**) germination rate 95%.

To investigate the intrinsic molecular mechanisms of radish seed dormancy, we first evaluated the phenotypic differences between seeds of the dormant variety (Rs275) and the non-dormant variety (Rs100). As shown in Figure 5, photographic records were captured against a contrasting dark background to ensure optimal visualization of radicle emergence and germination progress. After 24 h of germination, the non-dormant variety Rs100 exhibited high germination vigor, with germination rates of 98%, 99%, and 95% across three independent replicates. In contrast, germination of the dormant variety Rs275 was markedly inhibited, with germination rates of only 11%, 20%, and 12%. This pronounced divergence in germination phenotype provided a reliable physiological basis for subsequent gene expression analysis (Figure 5).

**Figure 6:**
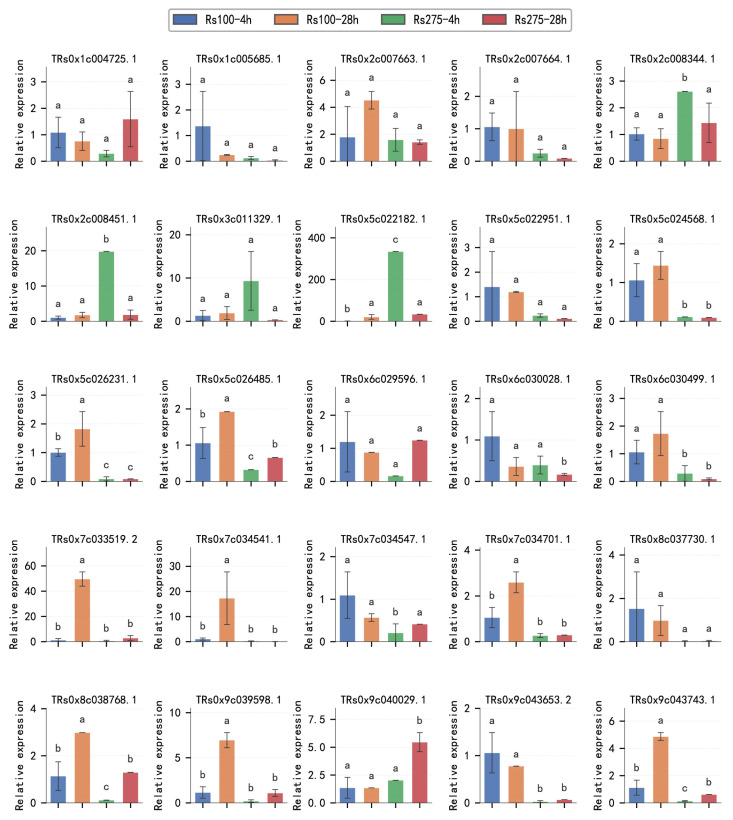
Dynamic qRT-PCR expression patterns of radish *RsDOG1* genes in dormant (Rs275) and non-dormant (Rs100) varieties. Different colored bars represent distinct combinations of radish cultivars and treatment time points (Rs100-4h, Rs100-28h, Rs275-4h, and Rs275-28h). Data are presented as mean ± SD of three biological replicates. Different lowercase letters (a–c) above the bars indicate statistically significant differences at the *p* < 0.05 level.

Based on the above phenotypic differences, quantitative real-time PCR (qRT-PCR) analysis (Figure 6) revealed the dynamic expression patterns of *RsDOG1* genes under different radish dormancy genotypes. The analysis found that three RsDOG1 genes *TRs0x2c008451.1*, *TRs0x3c011329.1*, and *TRs0x5c022182.1* exhibited remarkably high expression levels in dormant seeds at 4 h (Rs275-4h), whereas their expression was relatively low in non-dormant seeds (Rs100-4h). During the transition from 4 h to 28 h, key *RsDOG1* family members, particularly the highly expressed genes *TRs0x2c008451.1*, *TRs0x3c011329.1*, and *TRs0x5c022182.1*, showed a pronounced downregulation trend in expression in the dormant variety. This rapid response pattern is highly consistent with the degradation kinetics of the Arabidopsis *DOG1* gene after imbibition [12], suggesting that *RsDOG1s* may coordinately participate in the ‘switch’ regulation of the dormancy-to-germination transition in seeds. The low-level induced expression of certain genes in non-dormant varieties may imply that the *DOG1* gene family has undergone functional refinement and specific reprogramming during long-term domestication to adapt to diverse environmental conditions [15].

The expression amplitudes of individual genes showed significant divergence. Among them, *TRs0x5c022182.1* exhibited a relative expression level as high as approximately 340 in the dormant variety at 4 h (Rs275-4h), compared to less than 1.0 in the non-dormant variety (Rs100-4h), representing a 400-fold difference in expression. Concomitantly, the expression level of this gene decreased by over 90% at 28 h (Rs275-28h). These results indicate that *TRs0x5c022182.1* is likely a core orthologous candidate gene regulating seed dormancy in radish.

### 2.7. Intra-Species Collinearity Analysis of RsDOG1 Genes

**Figure 7:**
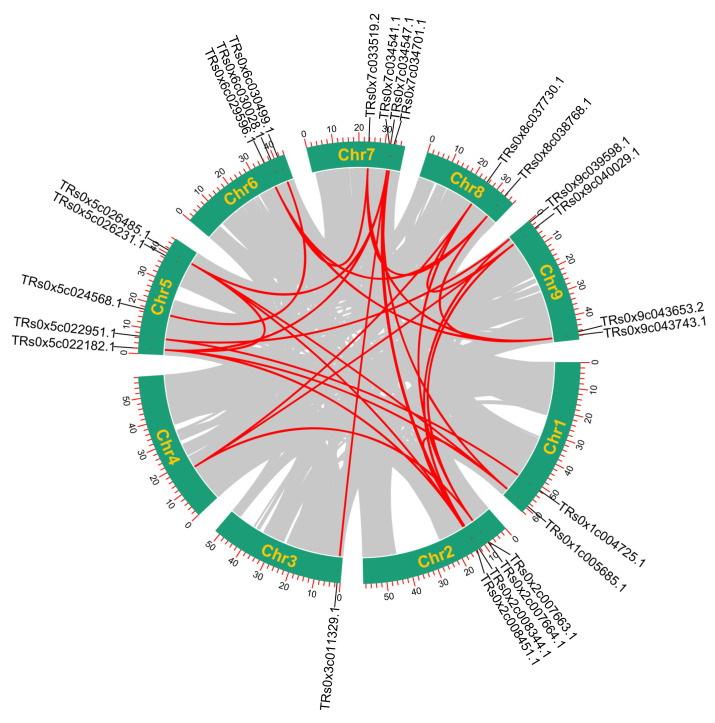
Intra-species collinearity analysis of the *RsDOG1* gene family in radish. Collinear gene blocks across the nine radish chromosomes (Chr1–Chr9) are shown as gray lines on the circular synteny map. Collinear gene pairs involving *RsDOG1* family members are highlighted in red. The chromosome-scale synteny map was generated using the Advanced Circos function in TBtools with MCScanX.

**Figure 8:**
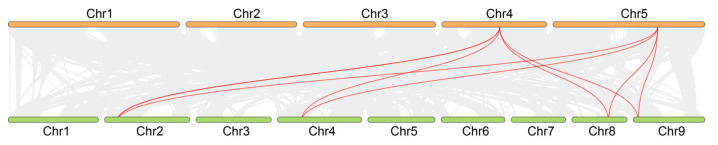
Inter-species collinearity analysis of *DOG1* genes between radish and *Arabidopsis thaliana*. Syntenic relationships between the radish genome (Chr1–Chr9) and the five *Arabidopsis* chromosomes are displayed. Gray lines represent genome-wide collinear blocks, while red lines specifically highlight syntenic *DOG1* orthologous gene pairs between the two species. The dual synteny plot was generated using TBtools.

To elucidate the evolutionary relationships, duplication patterns, and conservation of radish *DOG1* genes, both intra-genomic and cross-species synteny analyses were conducted. The intra-genomic analysis identified 28 collinear gene pairs across all nine chromosomes (Chr1–Chr9) (Figure 7), forming complex syntenic networks. For instance, *TRs0x2c008451.1* (Chr2) and *TRs0x1c005685.1* (Chr1) exhibited collinearity with three and four other *RsDOG1* genes, respectively, with Chr5 harboring the highest number of both inter- and intra-chromosomal pairings. This widespread distribution strongly indicates that segmental duplication was the predominant driver of *DOG1* family expansion in radish, consistent with observations in pepper [14] and *B. napus* [16]. Additionally, cross-species analysis revealed nine collinear pairs involving five *RsDOG1* genes (*TRs0x2c007664.1*, *TRs0x2c007663.1* on Chr2; *TRs0x4c017755.1* on Chr4; *TRs0x8c037730.1* on Chr8; and *TRs0x9c040029.1* on Chr9) and three *Arabidopsis DOG1* family members (*AT4G18650.1/AtDOG1*, *AT4G18660.1*, and *AT5G45830.1*) (Figure 8). Strikingly, all five radish genes displayed collinearity with the well-characterized seed dormancy regulators *AT4G18650.1* and/or *AT5G45830.1*, with *TRs0x2c007663.1* specifically showing collinearity with all three *Arabidopsis* members. These conserved syntenic relationships suggest functional conservation of *DOG1* family orthologs in regulating seed dormancy across Brassicaceae species, aligning with previous findings in peanut [17].

## 3. Discussion

In this study, 25 *RsDOG1* genes were identified in the radish genome. Physicochemical analysis revealed that RsDOG1 proteins are predominantly hydrophilic and unstable, implying tight post-translational degradation regulation during dormancy release [18,19]. Chromosomal localization showed clear physical clustering on Chr2 and Chr7, highlighting tandem duplication [6] as a primary driver of gene family expansion [9,20]. Furthermore, whole-genome collinearity analysis identified 28 intra-species collinear pairs, demonstrating that segmental duplication played a predominant role in expanding the *DOG1* family in radish, consistent with observations in pepper [14] and *B. napus* [16] during Brassicaceae evolution.

Phylogenetic analysis confirmed the high evolutionary affinity between radish and *Arabidopsis DOG1* genes [21] as dicotyledonous species. Notably, several RsDOG1 proteins harbor natural fusions with bZIP domains (bZIP superfamily or bZIP_HBP1b-like) [10,22]. In *Arabidopsis*, DOG1 controls dormancy primarily by binding and inhibiting PP2C phosphatases (e.g., AHG1 and AHG3) to activate downstream kinase cascades [18,19]. Because bZIP transcription factors regulate ABA signaling [23,24] and undergo antagonistic negative regulation by the gibberellin pathway [25], the fusion with bZIP domains may enable certain RsDOG1 proteins to directly enter the nucleus for transcriptional regulation [26], reflecting an evolved, more direct dual-function regulatory strategy in radish [27,28] for signaling integration.

Promoter analysis demonstrated that *RsDOG1* genes are highly enriched in ABREs, MYB-binding drought elements, anaerobic elements, and light-responsive elements, confirming their core roles in ABA-dependent seed dormancy [12,13] and environmental stress responses [14]. Divergent cis-element frequencies compared to wheat and peanut reflect evolutionary adaptation to specific microenvironments [5,8]. Extreme environmental fluctuations can significantly alter seed dormancy depth and trigger secondary dormancy [29,30]. The abundance of environmental response elements suggests that *RsDOG1* genes act as molecular hubs integrating internal hormone status with external climate changes [31,32] in response to field conditions.

Expression profiling suggests that *TRs0x5c022182.1* may represent a key potential candidate gene involved in regulating seed dormancy in radish, supported by conserved synteny with *Arabidopsis* dormancy regulators *AT4G18650.1 (AtDOG1)* and *AT5G45830.1*. Meanwhile, other members showing transient high expression in dormant seeds, such as *TRs0x2c008451.1* and *TRs0x3c011329.1*, might tentatively participate in early imbibition events or dormancy maintenance, though their precise regulatory roles remain to be further clarified. Conversely, the specific induction or higher expression of other *RsDOG1* genes (e.g., *TRs0x5c026231.1*, *TRs0x5c026485.1*, *TRs0x7c033519.2*, and *TRs0x9c043743.1*) in non-dormant cultivars or post-imbibition indicates significant functional divergence within the family [15]. These upregulated genes likely facilitate seed germination, metabolic mobilization, and radicle emergence. Cross-species studies further illuminate the roles of other *RsDOG1* members: *BnDOG1* in *Brassica napus* is expressed in roots and pollen and induced by manganese stress [16], while homologs in Moso bamboo (*Phyllostachys edulis*) and wheat (*Triticum aestivum*) are abundant in vegetative tissues responding to GA, ABA, and MeJA signaling [5,6]. Together with promoter stress-responsive elements (Figure 4) and bZIP domain fusions (Figure 3), these findings indicate that remaining *RsDOG1* genes act beyond seed dormancy, integrating phytohormone signaling and abiotic stress responses during vegetative growth [33]. Ultimately, these insights provide valuable genomic targets for molecular breeding to simultaneously optimize germination traits and environmental resilience.

Cross-species expression profiles suggest that other RsDOG genes function beyond dormancy, playing critical roles in vegetative environmental adaptation and stress mitigation. This expansion aligns with the bZIP domain fusion in many DOG1 proteins, which maintains redox homeostasis and activates pathways against heavy metal toxicity and oxidative damage. Additionally, predicted interactions in B. napus show DOG1 proteins bind stress-linked transcription factors (GRXC7, GRXC8, SCL14) that modulate detoxification networks under stress. Thus, *RsDOG1* family members act as versatile regulators integrating hormone signaling and stress responses during vegetative growth. This discovery fills a critical gap in radish seed dormancy mechanisms while providing solid targets for gene-editing crop improvement [33]. Ultimately, integrating structural and expression analyses broadens understanding of *DOG1* family evolution in Brassicaceae and provides valuable breeding resources for high-yield, pre-harvest sprouting-resistant radish varieties.

## 4. Materials and Methods

### 4.1. Identification of Radish DOG1 Gene Family Members

To identify *DOG1* gene family members in radish (*Raphanus sativus*), the whole-genome sequence and annotated protein database of radish (cultivar Xin-li-mei, CCHX genome assembly IVFCAAS_Rs00_1.1, NCBI GenBank assembly accession GCA_019703475.1; Available online: https://www.ncbi.nlm.nih.gov/datasets/genome/GCA_019703475.1/ (accessed on 20 July 2026) were obtained [21]. The reference sequence of *AtDOG1* was retrieved from the TAIR database [6]. Candidate *RsDOG1* genes were screened using a combined strategy of local Hidden Markov Model (HMM) search and local BLASTP against the radish protein database using TBtools [34]. Briefly, candidate proteins containing the conserved DOG1 domain were searched locally using the Pfam HMM profile (PF14144) [7] with an E-value threshold of 1 × 10^−5^. Concurrently, a local BLASTP search was performed against the local radish protein database using *Arabidopsis AtDOG1* as the query (*E*-value < 1 × 10^−5^) [35]. The results from both local search strategies were merged, and redundant sequences were removed. The remaining sequences were submitted to the InterPro database to validate domain integrity, and false-positive candidates lacking the core DOG1 domain were excluded, ultimately yielding 25 *RsDOG1* family members (Table S4). All gene identifiers (e.g., TRs0x…) presented in this study correspond to the original gene locus annotations from the published CCHX radish genome assembly database [21].

### 4.2. Physicochemical Property Analysis of Radish DOG1 Gene Family Members

Complete protein sequences of all the preliminarily identified radish *DOG1* gene family members were extracted and saved as a FASTA file. Physicochemical properties of all DOG1 proteins were predicted using the ExPASy ProtParam tool (SIB Swiss Institute of Bioinformatics, Geneva, Switzerland) [36] and TBtools software (v2.48) [34], including amino acid sequence length, molecular weight (MW), theoretical isoelectric point (pI), instability index (II), aliphatic index (AI), and grand average of hydropathicity (GRAVY) (Table S2).

### 4.3. Chromosomal Localization of Radish DOG1 Gene Family Members

To determine the physical positions and distribution characteristics of *DOG1* gene family members on radish chromosomes, the whole-genome sequence and corresponding GFF3/GTF genome annotation files of radish were obtained. After extracting the physical localization data of target genes, the gene list and annotation files were imported into TBtools software [34]. Gene localization information was generated using the built-in chromosome visualization module, and after parameter adjustments, high-resolution chromosomal distribution maps were exported.

### 4.4. Phylogenetic Analysis and Structural Characterization of the Radish DOG1 Gene Family

Conserved motifs and domain features of radish DOG1 proteins were predicted using the online MEME tool (MEME Suite, University of Washington, Seattle, USA)(maximum 10 motifs) [11] and the NCBI Conserved Domain Database (CDD), respectively. An intra-species phylogenetic tree was constructed using MEGA 11 [37] via the Maximum Likelihood (ML) method with the JTT substitution model and 1000 bootstrap replicates. The phylogenetic tree, motif composition, conserved domains, and exon–intron gene structures were integrated using TBtools [34]. For cross-species evolutionary analysis, amino acid sequences of DOG1 proteins from radish and *Arabidopsis* were aligned using MUSCLE (integrated into MEGA 11), and a Neighbor-Joining (NJ) tree was constructed in MEGA 11 [37] with 1000 bootstrap replicates (support values ≥ 50% shown). The resulting tree was visualized and edited using FigTree (v1.4.4) and TBtools [34] (Table S3).

### 4.5. Promoter Sequence Extraction and Cis-Acting Element Prediction of the Radish DOG1 Gene Family

To explore the potential transcriptional regulatory mechanisms of radish *DOG1* genes, cis-acting elements in their promoter regions were predicted and analyzed. First, based on the radish genome sequence and GFF/GFF3 annotation files, sequences 2000 bp upstream of the start codon (ATG) of all the identified radish *DOG1* genes were extracted using TBtools software. Subsequently, the processed positional information file was imported into the Simple BioSequence Viewer module of TBtools to visualize the distribution and positions of key cis-acting elements in the promoters of each radish *DOG1* gene [34].

### 4.6. Quantitative Real-Time PCR Analysis of the Radish DOG1 Gene Family

To validate the expression patterns of the *RsDOG1* gene family during radish seed dormancy and germination, two genotypes with contrasting dormancy characteristics—a dormant variety (Rs275) and a non-dormant variety (Rs100)—were selected for quantitative real-time PCR (qRT-PCR) analysis. These two contrasting cultivars were chosen from a preliminary screening of 122 radish accessions evaluated over a 7-day germination assay with three biological replicates per accession. Based on seed plumpness, germination percentage, and germination vigor, Rs275 was selected as the representative dormant variety (exhibiting relatively low germination percentage and vigor), while Rs100 was selected as the non-dormant variety (exhibiting high germination percentage and vigor).

Seeds were soaked in distilled water, and the first harvest was conducted after 4 h of imbibition. Following germination in darkness at 28 °C and 60% relative humidity in an illuminated incubator for 28 h, the second harvest was performed. Each sample was immediately frozen in liquid nitrogen and subsequently preserved at –80 °C in an ultra-low temperature freezer for later analysis. Three biological replicates were established for each cultivar, and 100 full and uniform seeds were chosen from each replicate. The specificity of all qRT-PCR primers was evaluated using the Primer Check (Simple e-PCR) module embedded in TBtools-II (v2.487).

Total RNA was extracted using the TRIzol method [38]. Approximately 100 mg of seed samples were ground to a fine powder in liquid nitrogen, and 1 mL of TRIzol reagent was added for total RNA extraction following the manufacturer’s instructions. RNA concentration and purity were assessed using a NanoDrop 2000 spectrophotometer (Thermo Fisher Scientific, Wilmington, DE, USA) (OD260/OD280 = 1.8–2.0), and RNA integrity was verified by 1% agarose gel electrophoresis. One microgram of total RNA was used for first-strand cDNA synthesis using a reverse transcription kit (PrimeScript RT reagent Kit with gDNA Eraser, Takara Biomedical Technology (Beijing) Co., Ltd.) according to the manufacturer’s protocol. The resulting cDNA was diluted 10-fold and used as the template for QRT-PCR.

Based on the radish genome sequence, specific quantitative primers for candidate *RsDOG1* genes were designed using Primer Premier 5.0 software, with primer lengths of 18–25 bp, annealing temperatures of 55–60 °C, and amplicon sizes of 100–250 bp (Table S1). QRT-PCR reactions were performed on a CFX96 Real-Time PCR Detection System (Bio-Rad, Hercules, CA, USA) using SYBR Green I fluorescent dye. The 20 µL reaction system contained 10 µL of 2× SYBR Premix Ex Taq II (TaKaRa), 0.8 µL each of forward and reverse primers (10 µM), 2 µL of cDNA template, and ddH2O to a final volume of 20 µL. The thermal cycling program was: 95 °C for 30 s; 40 cycles of 95 °C for 5 s and 60 °C for 30 s; followed by a melt curve analysis from 65 °C to 95 °C with a 0.5 °C increment every 5 s. Three technical replicates were performed for each sample.

The radish *GAPDH* gene was used as the internal reference gene [39], and relative expression levels of target genes were calculated using the 2^−ΔΔCt^ method [40]. Experimental data were statistically analyzed and plotted using Excel 2019 and GraphPad Prism 8.0. One-way analysis of variance (ANOVA) was performed for each gene across the four treatment groups, followed by Tukey’s honest significant difference (HSD) post hoc test for pairwise comparisons (*p* < 0.05). Significance groups were labeled using a compact letter display (*a*, *b*, *c*), where groups sharing the same letter are not significantly different. All the experiments were performed with three biological replicates, and the data are presented as mean ± standard deviation (SD). The primer sequences used for QRT-PCR are listed in Table S1.

### 4.7. Collinearity Analysis of Radish DOG1 Genes

To investigate the evolutionary mechanisms underlying the expansion of the *DOG1* gene family in radish (*Raphanus sativus* L.), both intra-species and inter-species collinearity analyses were performed. For intra-species analysis, the radish genome sequence (CCHX assembly) and its corresponding gene annotation file were used to construct a whole-genome synteny map using the One Step MCScanX tool implemented in TBtools (v2.0) [34,41]. Gene duplication events and syntenic relationships among the 25 identified *RsDOG1* genes were systematically examined, and collinear gene pairs involving *RsDOG1* members were extracted and highlighted. For inter-species analysis, collinearity between the radish genome and the *Arabidopsis thaliana* reference genome (TAIR10) was similarly analyzed using MCScanX within TBtools [34,41], allowing the identification of orthologous *DOG1* gene pairs between the two species. All the synteny maps were visualized using the Advanced Circos and Dual Synteny Plotter functions in TBtools [34,41]. This analytical approach was adopted from recent *DOG1* gene family studies in peanut [17], pepper [14], and *Brassica napus* [16].

## 5. Conclusions

In this study, we performed the first systematic genome-wide identification and comprehensive analysis of the *DOG1* gene family in radish, successfully identifying 25 *RsDOG1* family members harboring complete DOG1 domains. Through analyses of physicochemical properties, chromosomal distribution, and phylogenetic relationships, we confirmed the high conservation of radish *DOG1* genes during dicotyledonous plant evolution and revealed that tandem duplication is the core mechanism driving the expansion of this family. Notably, we discovered the natural fusion of certain RsDOG1 proteins with bZIP domains. Through comparative discussion with the classical PP2C-binding mechanism in Arabidopsis, we propose that this domain fusion may endow RsDOG1 proteins with the innovative function of directly entering the nucleus to act as transcription factors, thereby achieving more efficient transcriptional reprogramming of the ABA signaling pathway. Furthermore, the highly enriched environmental and hormone-responsive elements in the promoter regions highlight the pivotal role of *RsDOG1* genes in responding to climatic fluctuations and regulating secondary seed dormancy. Expression analysis further identified TRs0x5c022182.1 as the potential candidate gene that may be involved in seed dormancy regulation in radish. In summary, this study not only substantially broadens our understanding of the structural evolution and functional diversification of the *DOG1* gene family in Brassicaceae plants but also provides a solid theoretical foundation and superior gene targets for the future precision improvement of seed dormancy traits and stress tolerance in radish and other crops using gene editing technologies.

## Figures and Tables

**Table 1:** Physicochemical property analysis of radish *RsDOG1* gene family members.

Sequence ID	Number of Amino Acids	Molecular Weight	Theoretical pI	Instability Index	Aliphatic Index	Grand Average of Hydropathicity
TRs0x5c022182.1	332	36,886.39	8.99	53.47	75.66	−0.615
TRs0x5c026485.1	332	36,935.51	8.66	54.54	78.31	−0.603
TRs0x1c005685.1	335	37,329.07	8.66	50.8	79.04	−0.565
TRs0x7c034547.1	327	36,388.09	9.13	56.44	81.87	−0.523
TRs0x2c008451.1	326	36,631.05	8.87	62.3	73.71	−0.717
TRs0x9c043743.1	361	41,361.6	6.08	65.2	87.04	−0.584
TRs0x7c034701.1	366	41,995.4	6.61	44.45	74.1	−0.587
TRs0x2c008344.1	363	41,620.16	8.88	42.15	76.86	−0.558
TRs0x7c034541.1	449	50,061.46	6.9	63.79	79.35	−0.546
TRs0x1c004725.1	370	42,254.73	6.54	39.95	75.95	−0.54
TRs0x6c029596.1	385	43,721	5.98	59.46	75.04	−0.64
TRs0x5c022951.1	367	41,900.37	7.3	40.02	74.71	−0.546
TRs0x9c039598.1	389	44,378.26	7.78	44.48	80.75	−0.509
TRs0x7c033519.2	382	43,539.05	5.87	69.8	83.27	−0.56
TRs0x3c011329.1	454	50,462.69	6.7	59.78	79.34	−0.54
TRs0x5c024568.1	483	53,727.56	7.78	59.64	76.81	−0.49
TRs0x6c030499.1	799	87,192.69	6.78	52.82	86.55	−0.326
TRs0x8c038768.1	390	43,982.31	5.98	56.23	76.82	−0.565
TRs0x9c043653.2	441	49,681.86	5.94	50.15	78.12	−0.466
TRs0x2c007663.1	279	31,687.87	5.07	62.3	80.86	−0.516
TRs0x8c037730.1	290	33,065.45	5.13	60.2	88.79	−0.412
TRs0x9c040029.1	280	31,308.13	5.2	66.39	79.89	−0.564
TRs0x5c026231.1	237	27,195.47	5.74	53.46	88.02	−0.489
TRs0x2c007664.1	239	27,619.08	8.86	46.64	94.31	−0.187
TRs0x6c030028.1	397	44,772.51	6.05	48.7	81.34	−0.485

## Data Availability

The original contributions presented in this study are included in the article/Supplementary Material. Further inquiries can be directed to the corresponding authors.

## Supplementary Materials

The following supporting information can be downloaded at https://www.mdpi.com/article/10.3390/ijms27156761/s1.
